# Uptake of Generative AI Integrated With Electronic Health Records in US Hospitals

**DOI:** 10.1001/jamanetworkopen.2025.49463

**Published:** 2025-12-12

**Authors:** Jordan Everson, Paige Nong, Chelsea Richwine

**Affiliations:** 1 Office of the National Coordinator for Health Information Technology and Assistant Secretary for Technology Policy, US Department of Health and Human Services, Washington, DC; 2 Division of Health Policy & Management, University of Minnesota School of Public Health, Minneapolis

## Abstract

**Importance:**

There is widespread enthusiasm about generative artificial intelligence (AI), but no systematic evidence on its implementation across health care organizations.

**Objective:**

To describe adoption of generative AI integrated with the electronic health record (EHR) by nonfederal acute care hospitals, how adoption relates to experience using and evaluating predictive AI, and hospital characteristics.

**Design, Setting, and Participants:**

This survey study of nonfederal acute care US hospitals used the 2024 American Hospital Association (AHA) Information Technology (IT) Supplement survey. The survey was completed by individuals most knowledgeable about health IT at the participating hospitals.

**Exposures:**

Experience with predictive AI, source of predictive AI, local evaluation practices (evaluation for accuracy and bias as well as postdeployment evaluation), and EHR developer were collected from the 2024 AHA IT Supplement. Hospital characteristics, including critical access hospital status, multihospital system membership, and teaching status, were collected from the 2024 AHA Annual Survey. Hospital operating margins, uncompensated care burden, and percentage of discharges from Medicaid were collected from the 2022 Medicare Cost Report.

**Main Outcomes and Measures:**

Whether the hospital was an early adopter of generative AI integrated with their EHR (currently used generative AI), fast follower (planned to use in the next year), or delayed adopter (planned to use in 5 years, no plans, or do not know).

**Results:**

A total of 2174 hospitals (1003 [weighted percentage, 50.4%] small; 1382 [weighted percentage, 60.8%] urban core–based; 1668 [weighted percentage, 68.8%] part of a multihospital system) responded to questions about their use of AI (51.5% response rate). Overall, 762 hospitals (weighted percentage, 31.5%) were early adopters of generative AI in 2024, 540 (weighted percentage, 24.7%) were fast followers, and 872 (weighted percentage, 43.7%) were delayed adopters. In unadjusted analyses, independent hospitals and critical access hospitals were less likely to be either early adopters or fast followers than delayed adopters. In adjusted analyses, hospitals that used predictive AI were more likely to be early adopters or fast followers than delayed adopters (difference, 26.2 [95% CI, 16.8-35.6] percentage points). Users of Epic were more likely to be early adopters and fast followers than users of other EHRs (eg, likelihood of being an early adopter or fast follower, Epic vs Oracle users: 21.9 [95% CI, 16.3-27.4] percentage points). Hospitals that reported conducting all local evaluation practices (accuracy, bias, postdeployment) were slower to adopt than hospitals that reported only 1 evaluation practice (all local evaluation processes: 12.1 [95% CI, 4.5-19.6] percentage points less likely to be early adopters than fast followers).

**Conclusions and Relevance:**

In this survey study of US hospitals, more than half of US hospitals reported that they would likely implement generative AI by the end of 2025. Results indicate the value of providing support to ensure hospitals can adopt beneficial generative AI and the need for developing and disseminating best practices for generative AI evaluation across organizations.

## Key Points

**Question:**

Which US hospitals used generative artificial intelligence (AI) in 2024?

**Findings:**

In this survey study of 2174 nonfederal US hospitals, 31.5% reported using generative AI in 2024 and 24.7% planned to do so in 1 year. Independent hospitals and those with high Medicaid discharges were more likely to have no implementation plans, while those that reported robust local evaluation of predictive AI were more likely to plan to use generative AI in 1 year than to currently use it.

**Meaning:**

The findings of this study suggest that by the end of 2025, half of nonfederal acute care hospitals in the United States will use generative AI; there is evidence of a digital divide, whereby some hospitals adopt these tools without complete safeguards.

## Teaser

This survey study describes the current and planned implementation of generative artificial intelligence (AI) in nonfederal acute care US hospitals.

## Introduction

There is enormous enthusiasm for the potential impact of artificial intelligence (AI) on the delivery of health care.^1,2,3^ That enthusiasm is coupled with evidence-based concerns that AI is being used in situations where it is not accurate, where it is biased, or where its effectiveness is not well known.^4,5,6,7^ These concerns have led to policymakers simultaneously seeking to spur AI adoption and establish guidelines for responsible AI use, validation, and governance as well as broader calls for responsible AI.^8,9,10,11,12,13,14^

Although they are not mutually exclusive, 2 general types of AI are relevant to health care. Predictive AI leverages machine learning (ML) or other predictive models to identify patterns and predict the likelihood of certain outcomes (eg, readmission, disease onset, fall risk).^15^ More recently, generative AI, which creates novel text, image, audio, or related output has emerged as a tool in health care (including ambient scribes, billing code suggestions, and draft messages).^15,16^ A 2023 survey of 100 leading health care organizations indicated that 25% already used generative AI and that three-quarters were experimenting with or planning to scale generative AI.^17,18^ However, to our knowledge, there are no systematic national analyses of the use of generative AI in health care delivery across US hospitals.

There are widespread concerns that the performance of these tools in early validation studies may not reflect their use in practice.^19,20^ One strategy to address this concern and limit inaccuracies is to evaluate AI within health care organizations using their patients’ data.^4,20,21^ In 2023, slightly less than half of hospitals using predictive AI reported consistently conducting this kind of local evaluation, with lower rates of AI adoption and evaluation at independent hospitals compared with health systems.^22^ It is likely that some of the capabilities required to effectively validate predictive AI provide a foundation for monitoring generative AI, but the science of ensuring that generative AI tools are fair, appropriate, valid, effective, and safe is still emerging.^23,24^

Given the rapid pace of change in AI, we sought to describe uptake of generative AI by US hospitals and characterize differences in uptake across organizations. We also sought to assess whether hospital experience with predictive AI (use, development, and evaluation) was associated with uptake of generative AI. Indications that some hospitals are lagging in plans to implement generative AI may imply that the efficiency and benefits of this technology will be unevenly distributed. At the same time, if hospitals with little experience using and evaluating predictive AI are early adopters of generative AI, this may support concerns^25,26,27^ that rapid adoption, especially among this cohort, may lead to inaccurate, low-value, or even harmful use.

## Methods

In this survey study, we analyzed data from the 2024 American Hospital Association (AHA) Information Technology (IT) Supplement. The IT Supplement complements the AHA’s Annual Survey of hospitals, widely considered a census of US hospitals, by providing detailed data on hospital use of health IT. The IT Supplement was sent to chief executive officers of all US hospitals with instructions that the individual most knowledgeable about health IT at their hospital should complete the survey, which was fielded April to September 2024. This study was deemed exempt from institutional review board review by the Office of the Assistant Secretary for Technology Policy. Informed consent was not required as the survey collected information about organizations, not respondents. We followed American Association for Public Opinion Reporting (AAPOR) guidelines for reporting guideline for survey results, including reporting of survey questions and responses, weighting responses to reflect the population, and transparency in treatment of respondents with missing data and responses of “Don’t Know.”

The AHA Annual Survey includes 4376 nonfederal general acute care hospitals and is considered a census of these organizations. Overall, 2253 of these hospitals responded to the Supplement (51.5% response rate). We combined data related to hospital use of AI from the AHA IT Supplement with data on hospital characteristics from the AHA Annual Survey. Estimates were weighted to reflect the population of hospitals represented in the AHA Annual Survey using data available for all hospitals in the annual survey and estimating the probability that a hospital responded to the AHA IT Supplement based on those characteristics. Specifically, we used a logistic regression model, including hospital size, ownership, teaching status, system membership, cardiac intensive care unit, urban status, and region to estimate the probability of responding and then used inverse probability weights to increase the weight assigned to respondents that were estimated to have a low probability of responding so that weighted responses better reflect the national population of nonfederal acute care hospitals.

### Variables

#### Use of Generative AI

The primary outcome was hospitals’ reported use of generative AI. Respondents were asked whether their hospital or health system used a large language model (LLM) (eg, ChatGPT, GPT-4, Google Gemini, Nuance DAX) integrated into the electronic health record (EHR). We focused on AI integrated into the EHR to capture AI implemented by the organization and integrated into staff workflows. Borrowing from the Rogers adoption curve,^28^ we grouped hospitals’ use of generative AI into 3 categories: early adopters (respondents who indicated current use of LLMs integrated with their EHR), fast followers (respondents who indicated planned use of LLMs integrated with their EHR in the next year), and delayed adopters (respondents who reported plans to use LLMs in 5 years, no plans to use LLMs, or unsure).

#### Use of Predictive AI

Two survey questions asked about use of ML or other predictive models (ie, statistical regression and related approaches^29^) integrated with the EHR. Hospitals were also asked whether they applied predictive AI to 8 use cases (predicting health trajectories for inpatients, identifying high risk outpatients, monitoring health, recommending treatments, simplifying billing, facilitating scheduling, other operational processes, and other clinical use cases). We grouped responses into 4 mutually exclusive categories: (1) hospitals that used ML integrated with the EHR; (2) hospitals that used other predictive models integrated with the EHR but did not use ML models; (3) hospitals that used ML or other predictive models for any of the 8 use cases, but did not indicate that models were integrated with the EHR, which likely reflects a lower level of organizational and technical investment in the routine use of these models; and (4) hospitals that did not indicate that they used any predictive AI.

#### Local Evaluation of Predictive AI and Predictive AI Developers

To characterize hospitals’ experience evaluating AI, respondents were asked to indicate the proportion of predictive AI used at their hospital that had been evaluated using data from their hospital or health system for accuracy, for bias, and through postimplementation monitoring. For each type of evaluation, we created a binary variable capturing whether the hospital conducted that type of evaluation for most or all predictive AI and a count variable capturing whether the hospital reported none, 1, 2, or all 3 local evaluation activities. To describe the types of AI developers that hospitals relied on, respondents were asked to separately indicate whether they used predictive AI from each of three sources: their EHR developer, models they self-developed, and third-party developers.

#### Hospital Characteristics

We included several measures from the AHA Annual Survey to characterize differences in hospital use of generative AI, including critical access hospital status, rural location, multihospital system membership, hospital size (small, <100 beds; medium, 100-399 beds; and large, ≥400 beds), ownership status (for-profit, nonprofit, and government [state-, county-, or city-owned]), and teaching status (nonteaching hospital, resident program, and academic medical center). We included additional data on hospital resources from the 2022 Medicare Cost Report on hospital operating margins, uncompensated care burden, and percentage of discharges from Medicaid.

### Statistical Analysis

We estimated the weighted prevalence of hospitals’ use of generative AI and compared it with hospitals’ use of predictive AI integrated with their EHR. We then estimated the weighted prevalence of each category by hospital characteristics and experience with predictive AI. We used weighted bivariate multinomial logistic regression and postestimation means to calculate 95% CIs around the prevalence estimates.

Finally, we implemented 2 weighted logistic regressions estimating hospitals’ use of generative AI based on hospital characteristics and their use, evaluation, and developers of predictive AI. In the first regression, the binary outcome indicated whether the hospital was in the delayed adopter group relative to early adopter or fast follower groups. We calculated the predicted probabilities associated with each independent variable as well as the marginal effect (percentage point difference) of each variable on the likelihood a hospital was a delayed adopter relative to an early adopter or fast follower. Relative to presentation of odds ratios, this approach is intended to provide a straightforward measure of the association between dependent and independent variables in adjusted analysis.^30^

In the second regression, the binary outcome indicated whether the hospital was an early adopter relative to a fast follower. Positive coefficients associated with predictive AI experience would indicate that hospitals with more advanced use (measured by use of predictive AI integrated into the EHR) and evaluation of predictive AI were more likely to use generative AI. In contrast, negative coefficients would indicate that hospitals with less advanced use and evaluation of predictive AI were more likely to currently use generative AI. Statistical significance was assessed using a conventional threshold of *P* < .05. Analyses were conducted with Stata SE version 15 (StataCorp).

## Results

Representatives from 2174 hospitals responded to survey questions on AI. All percentages presented are weighted to reflect the population of nonfederal acute care hospitals. Overall, 1003 hospitals (50.4%) were small (Table 1), 875 (38.1%) were medium, and 296 (11.5%) were large; 1382 (60.8%) were in urban core–based statistical areas, while 792 (39.2%) were in rural areas; and 506 hospitals (31.2%) were independent, with 1668 (68.8%) part of multihospital systems.

**Table 1.** Unadjusted Associations Between Hospital Characteristics and Generative AI Adoption^a^

Characteristic	Weighted column % (95% CI)	Hospitals, weighted row % (95% CI)		
		Early adopter	Fast follower	Delayed adopter
Weighted %	100	31.5	24.7	43.7
Hospital size				
Small	50.4 (48.2-52.5)	26.8 (24.1-29.6)	18.8 (16.4-21.2)	54.4 (51.2-57.5)
Medium	38.1 (36.0-40.2)	34.4 (31.3-37.6)	30.1 (27.0-33.3)	35.4 (32.1-38.7)
Large	11.5 (10.2-12.8)	42.4 (36.7-48.1)	32.8 (27.3-38.3)	24.8 (19.6-29.9)
Teaching status				
Nonteaching	50.9 (48.7-53.0)	25.9 (23.3-28.6)	20.0 (17.5-22.5)	54.1 (50.9-57.2)
Minor teaching hospital	43.1 (41.0-45.2)	35.0 (32.0-38.0)	30.2 (27.2-33.1)	34.8 (31.8-37.9)
Major teaching hospital	6.0 (5.1-7.0)	53.9 (46.0-61.9)	25.9 (18.9-32.9)	20.2 (13.8-26.5)
Ownership				
Nonprofit	66.3 (64.2-68.5)	41.1 (38.6-43.6)	20.8 (18.8-22.9)	38.1 (35.6-40.6)
Government	20.1 (18.3-21.9)	16.9 (13.2-20.6)	19.6 (15.6-23.6)	63.5 (58.6-68.4)
For profit	13.6 (11.9-15.3)	6.5 (3.1-10.0)	51.4 (44.6-58.1)	42.1 (35.4-48.9)
Location				
Urban	60.8 (58.6-62.9)	35.5 (32.9-38.0)	28.9 (26.5-31.4)	35.6 (32.9-38.2)
Rural	39.2 (37.1-41.4)	25.4 (22.4-28.4)	18.2 (15.5-20.9)	56.4 (52.9-59.9)
Critical access status				
Non–critical access	70.2 (68.1-72.2)	34.7 (32.4-37.1)	28.5 (26.2-30.8)	36.8 (34.3-39.3)
Critical access	29.8 (27.8-31.9)	24.0 (20.6-27.4)	15.9 (12.9-18.8)	60.1 (56.1-64.1)
System affiliation				
System member	68.8 (66.6-70.9)	38.5 (36.1-40.8)	29.2 (26.9-31.4)	32.4 (30.1-34.7)
Independent hospital	31.2 (29.1-33.4)	16.3 (13.0-19.5)	15.0 (11.9-18.1)	68.8 (64.7-72.8)
Medicaid discharges				
Bottom 80%	79.7 (77.9-81.5)	33.3 (31.1-35.6)	24.1 (22.1-26.2)	42.5 (40.1-44.9)
Top 20%	20.3 (18.5-22.1)	24.4 (20.3-28.5)	27.1 (22.8-31.3)	48.6 (43.7-53.5)
Uncompensated care				
Bottom 80%	79.3 (77.5-81.1)	33.3 (31.1-35.5)	23.3 (21.2-25.3)	43.5 (41.0-45.9)
Top 20%	20.7 (18.9-22.5)	24.8 (20.8-28.8)	30.4 (26.0-34.8)	44.8 (40.0-49.7)
Operating margins				
Bottom 80%	79.9 (78.1-81.6)	30.7 (28.5-32.8)	21.2 (19.2-23.1)	48.2 (45.7-50.6)
Top 20%	20.1 (18.4-21.9)	34.9 (30.5-39.4)	38.8 (34.1-43.6)	26.3 (21.9-30.7)
Alternative payment model count				
0	35.8 (33.7-38.0)	19.4 (16.6-22.3)	28.0 (24.6-31.4)	52.6 (48.7-56.4)
1	15.7 (14.1-17.3)	20.4 (16.1-24.8)	19.1 (14.7-23.5)	60.5 (55.0-66.0)
2	19.3 (17.6-21.0)	38.1 (33.5-42.6)	19.8 (16.0-23.7)	42.1 (37.4-46.8)
3	19.5 (17.8-21.1)	44.5 (39.9-49.1)	27.3 (23.3-31.4)	28.2 (23.9-32.4)
4	9.7 (8.5-10.9)	55.0 (48.7-61.2)	26.3 (20.8-31.9)	18.7 (13.8-23.6)
EHR Developer				
Oracle	19.9 (18.2-21.7)	15.6 (12.0-19.1)	22.8 (18.8-26.8)	61.7 (56.9-66.4)
Epic	52.3 (50.1-54.4)	48.8 (46.0-51.7)	27.1 (24.6-29.7)	24.0 (21.5-26.5)
Meditech	15.8 (14.1-17.4)	14.3 (10.6-18)	33.9 (28.4-39.4)	51.7 (46.0-57.5)
Other	5.4 (4.3-6.5)	7.6 (2.6-12.5)	5.0 (1.1-10.3)	86.7 (80.1-93.3)
CPSI/Evident	6.6 (5.4-7.9)	3.0 (0.1-6.9)	5.0 (1.1-9.6)	91.2 (85.8-96.5)

Abbreviations: AI, artificial intelligence; EHR, electronic health record.

^a^ Percentages represent the weighted prevalence of each category by hospital characteristics and experience with predictive AI. Percentages and 95% CIs were estimated using weighted bivariate multinomial logistic regression and postestimation means to calculate 95% CIs around the prevalence estimates.

**Figure 1.**
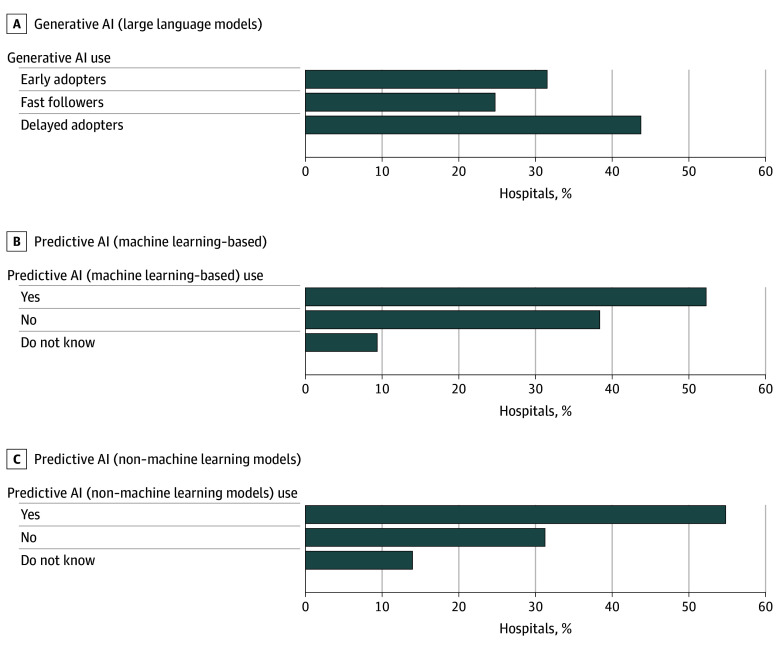
Hospital Reported Use of Predictive and Generative Artificial Intelligence (AI) Early adopters of generative AI are hospitals that indicated on the survey that they currently used a large language model integrated into the electronic health record. Fast followers are those planning to use generative AI in 1 year. Delayed adopters are those planning to use generative AI in 5 years (6.7%), with no plans to use generative AI (32.0%), and those who do not know (5.0%).

A total of 762 hospitals (31.5%) were early adopters of generative AI, meaning they indicated that their hospital or health system currently used generative AI integrated with their EHR (Figure 1). Another 540 hospitals (24.7%) were fast followers, indicating their hospital planned to use generative AI in the next year, and 872 hospitals (43.7%) were delayed adopters: 132 (6.7%) indicated plans in the next 5 years; 638 (32%) reported their hospital did not use generative AI and did not indicate plans to do so, and 102 (5%) did not know whether their hospital used generative AI. Overall, fewer respondents indicated their hospital currently used generative AI than used predictive AI (used by 1253 hospitals [52.2%]) or other predictive models (used by 1286 hospitals [54.8%]) integrated with the EHR.

### Unadjusted Association Between Hospital Characteristics and Use of Generative AI

Several hospital characteristics were associated with the use of generative AI (Table 1). For example, 53.9% (95% CI, 46.0%-61.9%) of major teaching hospitals were early adopters compared with only 25.9% (95% CI, 23.3%-28.6%) of nonteaching hospitals. Overall, 38.5% (95% CI, 36.1%-40.8%) of system-affiliated hospitals were early adopters, and an additional 29.2% (95% CI, 26.9%31.4%) were fast followers, compared with 16.3% (95% CI, 13.0%-19.5%) of independent hospitals that were early adopters and 15.0% (95% CI, 11.9%-18.1%) that were fast followers. Absolute differences in generative AI adoption by hospital characteristics were generally smaller than for predictive AI (eTable 1 in Supplement 1).

### Unadjusted Association Between Use of Generative AI and Predictive AI Experience

Hospital experience with predictive AI was strongly associated with use of generative AI (Table 2). For example, 52.8% (95% CI, 50.0%55.7%) of hospitals that used ML-based predictive AI were early generative AI adopters and 72.6% (95% CI, 65.5%-79.6%) of hospitals that did not use any predictive AI were delayed adopters. Additionally 47.7% (95% CI, 45.0%-50.4%) of hospitals that used predictive AI supplied by their EHR developer were early adopters of generative AI, whereas just 12.1% (95% CI, 8.6%-15.6%) of hospitals that did not use any predictive AI from their EHR developer were early adopters.

**Table 2.** Unadjusted Association Between Predictive Model Experience and Generative AI Adoption^a^

Characteristic	Weighted column % (95% CI)	Hospitals, weighted row % (95% CI)		
		Early adopter	Fast follower	Delayed adopter
Weighted %	100	31.5	24.7	43.7
Type of predictive AI used				
No predictive models	23.7 (21.8-25.7)	4.5 (2.5-6.4)	10.5 (7.6-13.4)	85 (81.6-88.4)
Only nonintegrated models	7.8 (6.6-9)	16.6 (10.5-22.7)	10.9 (6.2-15.5)	72.6 (65.5-79.6)
Non-ML predictive models integrated with EHR	16.2 (14.6-17.8)	9.7 (6.5-12.8)	65.4 (60.2-70.7)	24.9 (20.1-29.8)
ML-based predictive AI integrated with EHR	52.3 (50.1-54.4)	52.8 (50.0-55.7)	20.7 (18.4-23.0)	26.5 (24.0-29.0)
Among model users (n = 1752)	NA	40.0 (37.6-42.3)	29.2 (27.0-31.4)	30.9 (28.6-33.1)
Predictive AI model evaluation practices				
Most or all evaluated for accuracy	65.6 (63.3-67.9)	46.5 (43.6-49.4)	33.3 (30.5-36.1)	20.2 (17.9-22.5)
None, few, or some evaluated for accuracy	34.4 (32.1-36.7)	27.5 (23.8-31.1)	21.2 (17.8-24.6)	51.3 (47.1-55.6)
Most or all evaluated for bias	52.9 (50.5-55.3)	42.1 (38.9-45.3)	37.6 (34.4-40.8)	20.3 (17.7-22.9)
None, few, or some evaluated for bias	47.1 (44.7-49.5)	37.5 (34.1-40.9)	19.7 (16.9-22.5)	42.8 (39.2-46.4)
Most or all monitored postdeployment	53.3 (50.9-55.7)	40.4 (37.3-43.6)	39.4 (36.2-42.6)	20.2 (17.6-22.7)
None, few, or some monitored postdeployment	46.7 (44.3-49.1)	39.4 (36-42.8)	17.5 (14.8-20.2)	43.1 (39.5-46.7)
Count of predictive AI evaluation practices				
0 Practices	32.6 (30.3-34.9)	28.2 (24.4-32.0)	19.4 (16.0-22.8)	52.3 (48.0-56.7)
1 Practice	9.7 (8.3-11.1)	61.1 (53.8-68.3)	17.6 (12.1-23.1)	21.3 (15.1-27.6)
2 Practices	11.0 (9.5-12.4)	55.0 (48.1-61.9)	21.1 (15.3-26.9)	23.8 (18.0-29.7)
3 Practices	46.7 (44.3-49.1)	40.2 (36.8-43.6)	40.2 (36.8-43.7)	19.6 (16.9-22.3)
Predictive AI model source				
Self-developed models	48.2 (45.8-50.6)	42.9 (39.6-46.2)	29.8 (26.7-32.9)	27.3 (24.3-30.3)
Used no self-developed models	51.8 (49.4-54.2)	37.2 (34-40.5)	28.6 (25.5-31.7)	34.2 (30.9-37.5)
EHR developer models	78.2 (76.1-80.3)	47.7 (45-50.4)	22 (19.8-24.2)	30.3 (27.8-32.8)
Used no EHR developer models	21.8 (19.7-23.9)	12.1 (8.6-15.6)	55 (49.6-60.4)	32.9 (27.8-38.1)
Third-party models	50.6 (48.2-53)	38.9 (35.7-42.1)	38.2 (34.9-41.5)	22.9 (20-25.7)
Used no third-party models	49.4 (47-51.8)	41 (37.6-44.4)	19.9 (17.2-22.6)	39.1 (35.7-42.5)

Abbreviations: AI, artificial intelligence; EHR, electronic health record; ML, machine learning; NA, not applicable.

^a^ Percentages represent the weighted prevalence of each category by hospital characteristics and experience with predictive AI. Percentages and 95% CIs estimated using weighted bivariate multinomial logistic regression and postestimation means to calculate 95% CIs around the prevalence estimates.

### Adjusted Association Between Predictive AI Experience and Use of Generative AI

**Figure 2.**
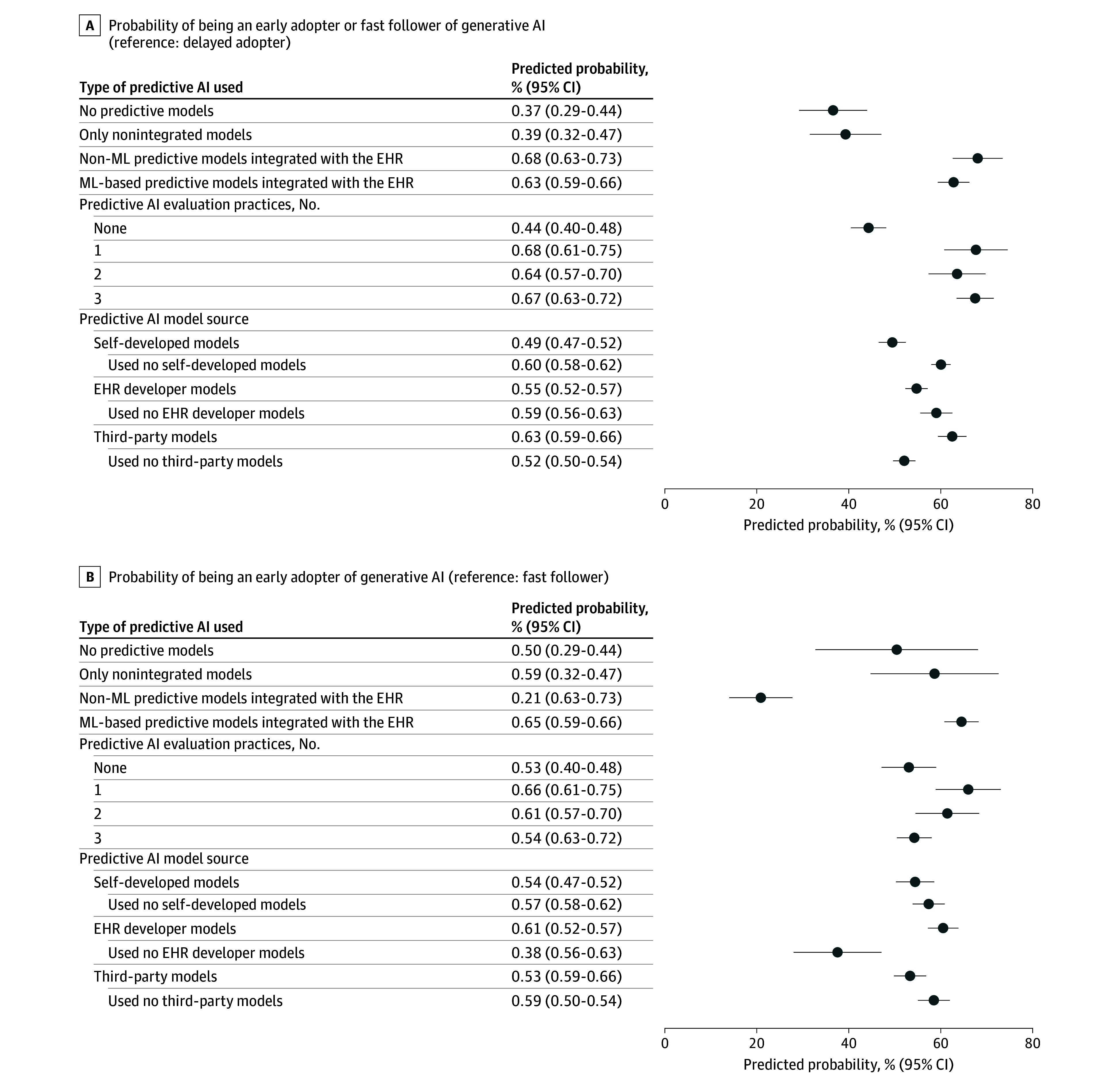
Predicted Probability of Generative Artificial Intelligence (AI) Adoption by Experience With Predictive AI Sample includes 2174 hospitals. Predicted probabilities were derived from multivariable logistic regression models that include hospital characteristics, reported in Table 3, to estimate associations with being a delayed adopter (no plans to use generative AI, plans to use generative AI within 5 years, or do not know) or not (A) and an early adopter (currently uses generative AI) as opposed to a fast follower (plans to use generative AI in the next year) (B).

In multivariate analysis, hospitals that used ML-based predictive AI integrated with the EHR were 26.2 (95% CI, 16.8-35.6) percentage points more likely to be early adopters or fast followers than hospitals that did not use predictive AI (Figure 2A and Table 3). Hospitals that reported 1 local evaluation practice for predictive AI were 23.3 (95% CI, 14.7-32.0) percentage points more likely to be early adopters or fast followers than hospitals that reported no evaluation practices.

**Table 3.** Marginal Effects of Hospital Characteristics on Generative AI Adoption

Characteristic	Difference (95% CI), percentage points^a^	
	Early adopter or fast follower (reference, delayed adopter) (n = 2174)	Early adopter (reference, fast follower) (n = 1302)
EHR Developer (reference, Oracle)		
Epic	0.219 (0.163 to 0.274)^b^	0.035 (−0.058 to 0.127)
Meditech	0.083 (0.012 to 0.155)^c^	0.086 (−0.018 to 0.190)
Other	−0.149 (−0.290 to −0.008)	0.130 (−0.099 to 0.359)
CPSI/Evident	−0.116 (−0.268 to 0.036)	0.111 (−0.153 to 0.375)
Hospital size (reference, small)		
Medium	0.005 (−0.042 to 0.052)	−0.026 (−0.092 to 0.039)
Large	−0.015 (−0.083 to 0.053)	−0.052 (−0.145 to 0.041)
Teaching status (reference, nonteaching)		
Minor teaching hospital	0.041 (−0.003 to 0.084)^d^	0.018 (−0.044 to 0.080)
Major teaching hospital	0.096 (0.012 to 0.180)^c^	0.097 (−0.007 to 0.200)^d^
Ownership (reference, nonprofit)		
Government	0.037 (−0.014 to 0.089)	−0.121 (−0.210 to −0.033)^b^
For profit	−0.025 (−0.093 to 0.042)	−0.236 (−0.384 to −0.088)^b^
Independent hospital (reference, multihospital system)	−0.021 (−0.072 to 0.029)	−0.012 (−0.093 to 0.070)
Rural location (reference, urban)	0.024 (−0.022 to 0.071)	0.006 (−0.062 to 0.074)
Critical access status (reference: non–critical access)	0.003 (−0.051 to 0.057)	−0.040 (−0.117 to 0.037)
Independent hospital (ref: multi-hospital system)	−0.021 (−0.072 to 0.029)	1.04 (0.66 to 1.66)
Count of alternative payment models	0.026 (0.012 to 0.040)^b^	−0.015 (−0.034 to 0.004)
Top 20% Medicaid discharges	−0.049 (−0.091 to −0.007)^c^	−0.089 (−0.151 to −0.027)^b^
Top 20% uncompensated care	−0.024 (−0.067 to 0.018)	−0.053 (−0.114 to 0.009)^d^
Top 20% operating margins	0.052 (0.005 to 0.099)^c^	0.059 (0.004 to 0.114)^c^

Abbreviation: AI, artificial intelligence.

^a^ Type of predictive AI used, predictive AI evaluation practices, and predictive AI model source were included in regression models presented here, with predicted probabilities reported in Figure 2. Coefficients derived from multivariable logistic regression models represent the marginal effect of each variable expressed as a percentage point increase in the likelihood that hospital with a given independent variable is either an early adopter (currently uses generative AI) or fast follower (plans to use generative AI in the next year) rather than a delayed adopter (no plans to use generative AI, plans to use generative AI within 5 years, or do not know) or not (data presented in second column) and an early adopter as opposed to a fast follower (data presented in third column).

^b^ *P* < .01.

^c^ *P* < .05.

^d^ *P* < .10.

Experience with predictive AI also strongly differentiated early adopters from fast followers (Figure 2B). Hospitals that reported all 3 local evaluation practices for most or all models were 12.1 (95% CI, 4.5-19.6) percentage points less likely to be early adopters than hospitals reporting 1 evaluation practice. Hospitals that reported using predictive AI from their EHR developer were 22.4 (95% CI, 11.0-33.9) percentage points more likely to be early adopters than hospitals that reported only using models from other sources. Results are presented as odds ratios in eTable 2 in Supplement 1.

### Adjusted Association Between Hospital Characteristics and Use of Generative AI

Several hospital characteristics were associated with generative AI use. Hospitals that used Epic were 21.9 (95% CI, 16.3-27.4) percentage points more likely than Oracle users (reference category) to be an early adopter or fast follower than a delayed adopter (Table 3), as were major teaching hospitals relative to nonteaching hospitals (9.6 [95% CI, 1.2-18.0] percentage points) and hospitals with high operating margins (5.2 [95% CI, 0.6-9.9] percentage points). Hospitals in more alternative payment models were also more likely to be early adopters or fast followers (2.6 [95% CI, 1.2-4.0] percentage points per additional alternative payment model). The 20% of hospitals with the highest proportion of Medicaid discharges were 4.9 (95% CI, 0.7-9.1) percentage points less likely to be early adopters or fast followers than hospitals with a lower proportion of Medicaid discharges.

A few characteristics also differentiated early adopters from fast followers. Hospitals with high operating margins were 5.9 (95% CI, 0.4 to 11.4) percentage points more likely to be early adopters (Table 3). For-profit and government hospitals were less likely to be early adopters than fast followers (for profit: −23.6 [95% CI, −38.4 to −8.8] percentage points; government: −12.1 [95% CI, −21.0 to −3.3] percentage points), as were hospitals with a high share of Medicaid discharges (−8.9 [95% CI, −15.1 to −2.7] percentage points).

## Discussion

In 2024, more than half of US hospitals reported either currently using (31.5%) generative AI integrated with their EHR or planning to do so in the next year (24.7%), representing rapid adoption in health care settings following the widespread emergence of generative AI in late 2022. Differences in generative AI adoption across hospital characteristics, primary EHR vendor, and experience evaluating predictive AI indicate that the digital divide observed with predictive AI may be replicated in the use of generative AI.^22^ In this national survey, hospitals that reported less robust evaluation of predictive AI were more likely to report being early adopters of generative AI, indicating that AI adoption may be driven more strongly by the hospital’s primary EHR developer and other resources to purchase AI tools rather than their capacity to locally evaluate and monitor AI to ensure its accuracy, fairness, and ongoing effectiveness.

Our results signal rapid adoption of generative AI in 2025, with more than half of US hospitals indicating they will implement generative AI integrated with the EHR by the end of the year. While few other data directly capture the use of generative AI, a 2024 survey found that 31% of family physicians reported using AI for clerical support, such as message drafting and ambient clinical documentation, and another survey of physicians found that 21% used AI for documentation.^31,32^ Our findings complement these by providing insight into the adoption of generative AI by hospitals rather than individual clinicians and by focusing on integration with the EHR, which likely represents an organization-level initiative to implement generative AI. This rapid adoption across the delivery system is likely driven both by an expectation that generative AI will address burnout among clinicians and increase patient volume and social, institutional, and mimetic processes.^33,34^

We observed the continuation of a digital divide across many hospital characteristics in adoption of generative AI.^22^ While there has been rapid adoption of generative AI at approximately one-quarter of hospitals, more than 30% reported no plans to implement generative AI. In unadjusted analysis, hospitals in multihospital systems were more than twice as likely to be early adopters of generative AI or fast followers than independent hospitals, and critical access and rural hospitals were substantially less likely to be early adopters or fast followers than their counterparts. These trends parallel previously reported digital divides in hospital adoption of health IT.^35,36^ However, this digital divide is harder to ascertain in adjusted analyses. This is likely because the associations between hospital characteristics and generative AI use is attenuated by correlations with 2 important factors: predictive AI experience and the hospital’s primary EHR developer.

Three factors differentiated early adopter, fast follower, and delayed adopter hospitals in multivariable analysis. First, hospitals that used predictive AI integrated with the EHR were substantially less likely to be delayed adopters of generative AI. This may reflect established organizational competencies and existing technological infrastructures that facilitate the adoption of generative AI.

Second, less than half of hospitals reported all model evaluation activities had been applied to the predictive AI they used, and almost one-third of hospitals indicated that none of these practices were followed. Hospitals that reported at least 1 of the 3 evaluation steps (evaluation for accuracy or bias or postimplementation evaluation) were less likely to be delayed adopters. This likely indicates that established competencies ensuring the effectiveness of AI are important to adopting new technology. However, we found that both hospitals reporting no local evaluation practices and hospitals reporting all 3 evaluation practices were less likely to be early adopters than hospitals that reported 1 local evaluation practice. This may indicate that fast followers can be thought of as 2 groups: (1) hospitals that have limited predictive AI evaluation experience and (2) those that have adopted a more cautious approach to AI as demonstrated by a track record of evaluating and monitoring all predictive AI models and are therefore somewhat slower to adopt generative AI. This second group is consistent with Rogers’ innovation diffusion framework, in that these hospitals likely possess the technical capacity to implement AI but require more robust evidence of success than early adopters.

Third, EHR developers appear central to the adoption of generative AI. Hospitals that used Epic were far more likely to be early adopters or fast followers than hospitals using other EHR developers. Hospitals that used predictive AI from their EHR developer were substantially more likely to be early adopters of generative AI than fast followers. These trends highlight the central importance of EHR developers in facilitating adoption of generative AI that is readily available through integration with the EHR, and in particular the central importance of Epic, the market-leading developer by a substantial margin. The strength of these associations, combined with the substantial share of hospitals that use the 3 largest vendors in our data, may indicate that a handful of EHR developers have substantial ability to influence competition among health AI firms. The apparent reliance on EHR developers, combined with the majority of hospitals reporting incomplete local evaluation and monitoring of predictive AI, might raise concerns that some hospitals that have rapidly adopted generative AI are not well-equipped to participate in governance of AI.^37,38,39^ This dynamic increases the importance of EHR developers and other AI developers acting to ensure the AI that they provide their customers is effective and safe as health systems continue to build the capacity to participate in governance.

### Limitations

Our study is subject to several limitations. First, it is cross-sectional and descriptive in nature, and the associations do not indicate causation. Nevertheless, the differences we observed point to important divides. Second, the outcome captures only the use of generative AI integrated in the EHR, which may not fully capture certain administrative applications of the technology. Crucially, the outcome variable does not capture specific applications of generative AI (eg, responding to patient messages, ambient scribes). It is likely that our results are driven by a few high-profile use cases, including ambient scribes and perhaps message drafting tools. These results therefore indicate the prevalence of adoption of generative AI at US hospitals but do not capture the intensity or diversity of use, which may be growing. Further attention to use-case specific uptake of generative AI will be important follow-up work.

## Conclusions

In this national survey study of US hospitals, nearly one-third of US hospitals had adopted generative AI integrated with their EHR in 2024, with another quarter planning to implement it in the next year. Hospitals with limited experience using predictive AI were less likely to adopt generative AI, while fast followers reported more AI evaluation practices than early adopters. Hospitals that reported Epic as their primary EHR were far more likely to be early adopters or fast followers in generative AI, indicating that hospitals may be relying on their EHR systems to facilitate AI use. Pairing rapid adoption of generative AI with rapid development and dissemination of best practices to effectively evaluate, monitor, and improve generative AI will likely be essential to ensuring long-term value from the technology.

## Notes

Supplement 1. **eTable 1. **Tabulation of Predictive AI Use and Evaluation with Hospital Characteristics **eTable 2.** Logistic Regression of Predictive AI Experience and Hospital Characteristics on Generative AI Adoption

Supplement 2.
Data Sharing Statement
