# Supplementary material for: Uptake of Generative AI Integrated With Electronic Health Records in US Hospitals
Source: JAMA Netw Open. 2025 Dec 12;8(12):e2549463. doi: 10.1001/jamanetworkopen.2025.49463 (PMC12701511; doi:10.1001/jamanetworkopen.2025.49463)
Supplement: Supplement 1. — eTable 1. Tabulation of Predictive AI Use and Evaluation with Hospital Characteristics eTable 2. Logistic Regression of Predictive AI Experience and Hospital Characteristics on Generative AI Adoption [file jamanetwopen-e2549463-s001.pdf]

Supplemental Online Content

Everson J, Nong P, Richwine C. Uptake of generative AI integrated with electronic health records in US hospitals. *JAMA Netw Open.* 2025;8(12):e2549463. doi:10.1001/jamanetworkopen.2025.49463

eTable 1. Tabulation of Predictive AI Use and Evaluation with Hospital Characteristics

eTable 2. Logistic Regression of Predictive AI Experience and Hospital Characteristics on Generative AI Adoption

This supplemental material has been provided by the authors to give readers additional information about their work.

© 2025 Everson J et al. *JAMA Network Open*.

eTable 1. Tabulation of Predictive AI Use and Evaluation with Hospital Characteristics

	Used predictive AI or other predictive models integrated into EHR (N=2,174)	Evaluated models for both accuracy and bias (N=1,572)
	Percent	Percent
**Hospital Size (Ref: Small)**	55.6%	51.6%
Medium	78.1%	60.8%
Large	92.7%	61.0%
**Teaching Status (Ref: Non-teaching)**	56.9%	52.7%
Minor Teaching Hospital	79.0%	59.6%
Major Teaching Hospital	90.1%	64.3%
**Ownership (Ref: Non-Profit)**	76.9%	55.3%
Government	40.8%	42.6%
For-Profit	68.0%	79.6%
Urban Location	78.4%	60.4%
Rural Location	53.0%	49.4%
Non-Critical Access	47.2%	59.6%
Critical Access Status	77.5%	47.4%
System Member	83.2%	61.5%
Independent Hospital	36.0%	33.9%
Bottom 80% Medicaid Discharges	68.2%	58.0%
Top 20% Medicaid Discharges	69.3%	53.4%
Bottom 80% Uncompensated Care	68.6%	55.4%
Top 20% Uncompensated Care	67.7%	63.5%
Bottom 80% Operating Margins	64.2%	53.4%
Top 20% Operating Margins	85.4%	67.8%

N=2,147 hospitals. All reported percents are weighted for non-response as described in the Methods.

© 2025 Everson J et al. *JAMA Network Open*.

eTable 2. Logistic Regression of Predictive AI Experience and Hospital Characteristics on Generative AI Adoption

	Early Adopter / Fast Follower (Ref: Delayed Adopter)		Early Adopter (Ref: Fast Follower)	
	Odds Ratio	95% CI	Odds Ratio	95% CI
**Type of Predictive AI Used (Ref: No Predictive Models)**				
Only Non-Integrated Models	1.16	(0.65 - 2.06)	1.50	(0.55 - 4.09)
Non-ML Predictive Models Integrated with EHR	5.66[a]	(3.33 - 9.63)	0.21[a]	(0.08 - 0.57)
ML-based Predictive AI Integrated with EHR	4.15[a]	(2.57 - 6.71)	1.93	(0.80 - 4.65)
**Count of Predictive AI Evaluation Practices (Ref: 1 Practice)**				
0 Practices	0.26[a]	(0.16 - 0.43)	0.44[a]	(0.24 - 0.80)
2 Practices	0.78	(0.46 - 1.32)	0.78	(0.42 - 1.45)
3 Practices	0.99	(0.64 - 1.54)	0.47[a]	(0.29 - 0.78)
**Predictive AI Model Source**				
Self-develop models (Ref: Used models from other sources only)	0.47[a]	(0.34 - 0.64)	0.84	(0.58 - 1.21)
EHR Developer Models (Ref: Used models from other sources only)	0.74	(0.52 - 1.06)	3.22[a]	(1.84 - 5.65)
Third-Party Models (Ref: Used models from other sources only)	1.95[a]	(1.50 - 2.52)	0.78	(0.57 - 1.06)
**EHR Developer (Ref: Oracle)**				
Epic	3.46[a]	(2.55 - 4.70)	1.22	(0.72 - 2.08)
Meditech	1.58[b]	(1.06 - 2.35)	1.68	(0.89 - 3.15)
Other	0.43[b]	(0.18 - 1.00)	2.25	(0.47 - 10.78)
CPSI/Evident	0.52	(0.22 - 1.26)	1.98	(0.34 - 11.50)
**Hospital Size (Ref: Small)**				
Medium	1.04	(0.76 - 1.42)	0.86	(0.58 - 1.27)
Large	0.91	(0.58 - 1.43)	0.74	(0.43 - 1.27)
**Teaching Status (Ref: Non-teaching)**				
Minor Teaching Hospital	1.31[c]	(0.98 - 1.73)	1.11	(0.77 - 1.60)
Major Teaching Hospital	1.91[b]	(1.07 - 3.41)	1.82+	(0.93 - 3.57)
**Ownership (Ref: Non-Profit)**				
Government	1.29	(0.90 - 1.87)	0.52[a]	(0.32 - 0.82)
For-Profit	0.84	(0.54 - 1.32)	0.28[a]	(0.13 - 0.61)
Independent Hospital (Ref: Multi-hospital system)	0.87	(0.62 - 1.21)	0.93	(0.58 - 1.50)
Rural Location (Ref: Urban)	1.18	(0.85 - 1.63)	1.04	(0.69 - 1.55)
Critical Access Status (Ref: Non-Critical Access)	1.02	(0.71 - 1.47)	0.79	(0.51 - 1.24)
Top 20% Medicaid Discharges	0.72[b]	(0.55 - 0.95)	0.60[a]	(0.43 - 0.85)

© 2025 Everson J et al. *JAMA Network Open*.

Top 20% Uncompensated Care	0.85	(0.64 - 1.13)	0.74[c]	(0.52 - 1.05)
Top 20% Operating Margins	1.42[b]	(1.04 - 1.95)	1.43[b]	(1.01 - 2.02)
Count of Alternative Payment Models	1.19[a]	(1.08 - 1.31)	0.91	(0.82 - 1.02)
Number of Hospitals	2,174		1,302	

[a] $p<0.01$; [b] $p<0.05$; [c] $p<0.10$

Odds ratios derived from multivariable logistic regression models and represent the relative odds that hospital with a given independent variable is (1) either an early adopter (currently uses generative AI) or fast follower (plans to use generative AI in the next year) rather than a delayed adopter (no plans to use generative AI, plans to use generative AI within 5 years, or don't know) or not and (2) an early adopter as opposed to a fast follower.

© 2025 Everson J et al. *JAMA Network Open*.
