# Supplementary material for: Uptake of Generative AI Integrated With Electronic Health Records in US Hospitals
Source: JAMA Netw Open. 2025 Dec 12;8(12):e2549463. doi: 10.1001/jamanetworkopen.2025.49463 (PMC12701511; doi:10.1001/jamanetworkopen.2025.49463)
Supplement: Supplement 2. — Data Sharing Statement [file jamanetwopen-e2549463-s002.pdf]

## Data Sharing Statement

Everson. Uptake of Generative AI Integrated With Electronic Health Records in US Hospitals. *JAMA Netw Open*. Published December 12, 2025. doi:10.1001/jamanetworkopen.2025.49463

**Data**
**Data available:** Yes
**Data types:** Data (not involving human participants)
**How to access data:** Data is available for purchase from the American Hospital Association.
**When available:** beginning date: 05-01-2025

**Supporting Documents**
**Document types:** None

**Additional Information**
**Who can access the data:** Available for purchase.
**Types of analyses:** For discussion with the American Hospital Association.
**Mechanisms of data availability:** Purchasable from the AHA.
